# The Impact of Alkaloid-Producing *Epichloë* Endophyte on Forage Ryegrass Breeding: A New Zealand Perspective

**DOI:** 10.3390/toxins13020158

**Published:** 2021-02-18

**Authors:** Colin Eady

**Affiliations:** Barenbrug, New Zealand Ltd., 2547 Old West Coast Road, Courtenay, Christchurch 7671, New Zealand; ceady@barenbrug.co.nz; Tel.: +64-27-717440

**Keywords:** endophyte transmission, livestock safety, insect testing, quality control, alkaloid profile

## Abstract

For 30 years, forage ryegrass breeding has known that the germplasm may contain a maternally inherited symbiotic *Epichloë* endophyte. These endophytes produce a suite of secondary alkaloid compounds, dependent upon strain. Many produce ergot and other alkaloids, which are associated with both insect deterrence and livestock health issues. The levels of alkaloids and other endophyte characteristics are influenced by strain, host germplasm, and environmental conditions. Some strains in the right host germplasm can confer an advantage over biotic and abiotic stressors, thus acting as a maternally inherited desirable ‘trait’. Through seed production, these mutualistic endophytes do not transmit into 100% of the crop seed and are less vigorous than the grass seed itself. This causes stability and longevity issues for seed production and storage should the ‘trait’ be desired in the germplasm. This makes understanding the precise nature of the relationship vitally important to the plant breeder. These *Epichloë* endophytes cannot be ‘bred’ in the conventional sense, as they are asexual. Instead, the breeder may modulate endophyte characteristics through selection of host germplasm, a sort of breeding by proxy. This article explores, from a forage seed company perspective, the issues that endophyte characteristics and breeding them by proxy have on ryegrass breeding, and outlines the methods used to assess the ‘trait’, and the application of these through the breeding, production, and deployment processes. Finally, this article investigates opportunities for enhancing the utilisation of alkaloid-producing endophytes within pastures, with a focus on balancing alkaloid levels to further enhance pest deterrence and improving livestock outcomes.

## 1. Introduction

Efficiently producing animal-sourced food (ASF) from pasture using ‘low-cost livestock’ (in situ grazing of forage) is a key requirement in order meet land-use sustainability criteria [1]. New Zealand pastoral farming systems are some of the most efficient ‘low-cost livestock’ systems in the world, producing ASF economically and with a relatively small environmental footprint [2,3,4], and recently https://www.dairynz.co.nz/news/research-shows-nz-dairy-the-world-s-most-emissions-efficient/ accessed on: 11 February 2021. This is principally because the NZ climate is conducive to high year-round biomass production of high metabolizable energy, palatable temperate pasture. *Lolium perenne*, ryegrass, although an introduced species, is currently the principle grass of choice in NZ because cultivars have been bred to perform within NZ climatic ranges [5], with a cardinal temperature that enables good growth [6], along with architecture and physiology to provide high year-round photosynthetic conversion efficiency [7]. To complement these plant characteristics, management requires the addition of low-cost nutrients provided in part by addition of a legume, white clover, nitrogen and phosphate [8]. Persistence (biomass production/time) of such pastures is achieved through a combination of good stock and pasture management, breeding of the ryegrass germplasm, e.g., for rust resistance, seasonal growth, and the use of *Epichloë* endophytes to relieve biotic and abiotic stressors. These endophytes are asexual, maternally inherited and produce characteristic alkaloid profiles [9]. The compatibility of endophytes with the grass host, the effect they have on the host phenotype, and the temporal, spatial alkaloid profile produced are key components that need to be understood before deployment decisions can be made. The successful use of *Epichloë* endophytes has been achieved despite numerous application problems, and no single perfect endophyte exists on the market. Biologically, this is not surprising as the asexual *Epichloë* is trapped in the vegetative tissue or seed of a host ecotype. Variants are essentially limited to somatic mutations as recombination and reassortment of chromosomes do not occur. There are exceptions to this and hybridisations between endophytes have occurred throughout their evolution [10,11] to produce diploid and triploid interspecific hybrids. For more on the evolution of endophytes, refer to the works of Schardl and Hettiarachchige [9,10,12]. These biological characteristics favour the development of local variants evolved to live within a geographically constrained grass ecotype with specific growth characteristics, e.g., architecture, flowering dates, dormancy, and under local biotic, and abiotic stressors. Endophyte discovery projects [13] have found localised variants such as AR37, nea2, nea6, and CM142. Transfer of endophytes between ecotypes or species can cause gross changes to the symbiosis [13]. Elite forage ryegrass has been bred, through sexual recombination, primarily for biomass production and performance within an environment. This may rapidly change architecture, heading date, vernalisation requirement, etc., of the plant, which a recent study has suggested may limit the symbiotic association [14]. More studies are required to fully understand what dictates the temporal and spatial growth of the endophyte in the ryegrass and particularly the related alkaloid expression profiles. Many studies have been undertaken that demonstrate host-induced differences in *Epichloë* traits, aside from transmission and viability that are crucial production traits. The same *Epichloë* in a different ryegrass host can demonstrate an >10-fold alkaloid expression range [15] and/or have altered infection characteristics. Previous research has primarily been one-off studies, comparing one combination with another [15]. Trying to exactly copy biotic conditions, sampling, analysis, etc., to compare between manuscripts is difficult, thus making it difficult to home in on the causation(s) of any differences. One theme that comes through is that tetraploid ryegrass tends to have lower alkaloid concentrations than diploid, perhaps not surprising as the larger cell size of the tetraploids would provide a larger plant cell volume: endophyte ratio, thus diluting any endophyte contribution. A better understanding of the interplay between host and endophyte is required and until then, each ryegrass cultivar–*Epichloë* strain relationship must be assessed on its own merits.

This manuscript provides a brief overview of endophyte strains used in New Zealand and the ergot profiles produced, and then focuses on the practical breeding of ryegrass containing *Epichloë* endophyte and outlines some of the key challenges facing a grass seed company’s day to day breeding, production, and distribution. This manuscript will highlight quality control (QC) testing requirements and their difficulties, and functionality testing requirements of the industry, including shortfalls in alkaloid knowledge. This manuscript then investigates opportunities for breeding the host to better accept an endophyte as well as advances in endophyte selection, and deployment systems for use in future pasture systems. These highlights will be made whilst focused on *Epichloë*–ryegrass relationships in NZ, the issues and opportunities outlined are likely applicable to endophytes of other crop and forage species.

## 2. Brief History of Endophyte Strains Used in New Zealand Grass Breeding

Endophytic fungi have been known about for a long time [16] but the connection to animal livestock health and insect deterrence took some time to discover. It was not until the late 1970s in the USA that an endophytic fungus in tall fescue (*Festuca arundinacea*) was shown to cause fescue toxicosis in cattle [17] and then in New Zealand in the early 1980s the closely related endophyte in perennial ryegrass was shown to cause ryegrass staggers in sheep [18]. In the subsequent discovery period and following a series of name changes, they eventually became known as *Epichloë festucae* var. *lolii* and the tall fescue endophyte *Epichloë coenophiala* [19]. The original New Zealand var. *lolii* strain that produces alkaloids peramine, lolitrem B and ergovaline was termed Standard Endophyte (SE), wild type or Common Toxic to distinguish it from the strains discovered from there on. Endosafe™ was the first commercial ryegrass endophyte, which was released in 1990. From the literature, it appears (but is difficult to confirm) that Endosafe™ was originally AR6, which itself was two strains, later identified to be AR5 and AR77, which both produce peramine and ergovaline [20,21]. Initial studies (1991) indicated that Endosafe™ demonstrated good insect deterrence and was safe for animal performance [22]. This claim was quickly questioned as subsequent studies gave animal health issues attributed to the ergovaline [21,23] and it became understood that alkaloid profiles of the same endophyte could differ substantially within different hosts [15]. Endosafe™ in diploid perennial ryegrass cultivar ‘Pacific’ was withdrawn from the market, but the tetraploid cultivar ‘Greenstone’ with Endosafe™ continued to be sold. Reselection (possibly from the original Endosafe™) for lower ergovaline identified the strain AR5 which was later marketed as Endo5 and is still sold in Australia today. The known role of ergot alkaloids in fescue toxicosis steered ryegrass endophyte research away from ergot alkaloid-producing *Epichloë* and, shortly after, Endosafe™ strain AR1 was introduced to the New Zealand market in 2001. AR1 produced peramine but no ergovaline or lolitrems and proved to be animal safe and demonstrated good Argentine stem weevil deterrence [24,25,26], leading to its successful uptake by the NZ pastoral sector. Unfortunately, the single alkaloid profile of AR1 did not deter black beetle and other pests, which led to widespread product failure in the upper North Island of NZ, where these pests are prevalent [27].

AR1 was the first endophyte to obtain plant variety rights (PVR) protection in NZ, which set the precedent for future endophytes (Table 1). After AR1, the NEA2 endophyte in diploid cultivar ‘Tolosa’ was released. This endophyte was initially not fully characterised (see below) and produced moderate levels of peramine and ergovaline, and low levels of lolitrems. The product was withdrawn due to seed production issues. In 2007, a fourth ryegrass endophyte, AR37, was released to the market. AR37 ryegrass appeared to have similar or better persistence than ryegrass with SE endophyte [28,29]. AR37 still caused occasional outbreaks of ryegrass staggers, which could be severe but generally animal production compared favourably with nil endophyte ryegrass [30]. AR37 produces epoxy-janthitrem alkaloids, via a similar biochemical pathway to that for lolitrem production, but janthitrems have a lower potency than lolitrem B [31,32]. Again, as with Endosafe, it was shown that the individual host–endophyte relationship was important in regulating alkaloid expression with some AR37/ryegrass cultivars causing greater staggers than others (certain AR37 products contain warnings to this effect). In some studies, a decrease in milk solids production in dairy cows has also been observed in pastures containing AR37 [26,33]. License terms for AR37 caused some NZ companies to search for alternative endophytes. This led to additional Novel Endophyte Agriseed (NEA) endophytes being developed. These have primarily originated in Spanish ryegrass germplasm but, as the discovery programme gained momentum endophytes from other regions of the world have been included. Many hundreds of *Epichloë* were screened but present commercial NEA endophytes are derived mainly from three strains—nea2, nea6, and nea3. Note most endophyte strains are denoted by capital letter(s) and a number, for the sake of clarity the ‘nea’ strains are represented here by lowercase to distinguish them from the commercial ‘NEA’ products. The nea strains are marketed singularly or in combination as NEA, NEA2, or NEA4; and produce a combination of peramine, ergovaline and a low level of lolitrem B. They have been tested to be animal safe and give good insect deterrence, but like AR37, host–endophyte interactions are important and industry evaluation tables carry caveats for animal performance issues under extreme circumstances (https://www.nzpbra.org/ accessed on: 11 February 2021). Ryegrass containing AR37 or NEA endophytes have been the principle proprietary perennial or hybrid ryegrasses sold in NZ over the last 10 years. Other companies have followed, either through licensed products or by discovering their own endophytes, although commercial success in the market has been limited. CropMark released U2 a *N*-formylloline-producing meadow fescue endophyte (*Epichloë uncinatum*) and it appears that they have tried to aid stabilisation of this in ryegrass through creation of festulolium hybrids. Recently, they have protected a new endophyte CM142 (NZ PVR website) classed as a novel janthitrem-producing *Epichloë*. DLF (DLF.com) market Edge, an *Epichloë festucae* var *lolii* that produces high peramine, low lolitrem B and ergovaline (potentially similar to nea2); and an *Epichloë coenophiala* called Protek that produces low ergovaline and loline, and is derived from (and for) tall fescue (2017 Australian plant breeders rights); and an *Epichloë siegelii* called Happe, a meadow fescue endophyte from Germany that produces lolines and is suitable for use in some ryegrass offering protection against porina [34]. Meanwhile, AgResearch extended their species range through the discovery of AR501, a non-ergovaline-producing tall fescue endophyte, which they superseded with AR542 and AR584 and market as MaxP and MaxQ [35] for use in fescue, and Barenbrug (barusa.com) came out with a similar product E34^®^. Although a couple of different *Epichloë* species above are being sold in ryegrass (U2 and Happe), it is generally recognised that switching host species for an endophyte is difficult and usually results in gross symbiotic changes that render the relationship unmarketable [13]. Whilst recognising these other host–*Epichloë* relationships, this review focusses on *Epichloë festucae* var *lolii* in ryegrass.

The use of *Epichloë* endophytes as a ‘trait’ of the plant is an almost unique characteristic of forage grass plant breeding. Its commercial success is demonstrated by expansion of its first designed use in New Zealand to Australia, South America, and South Africa. Development of *Epichloë* for Fescues, for example MaxQ and MaxQII [35], has further extended the use to North American markets. There is also a renewed interest in Europe for the use of endophytes as biological control agents due to increasing constraints on synthetic chemical use. AgResearch has valued the contribution of endophytes to be worth $200 million NZD (~118M Euro) a year to the NZ economy [36]. The AR37 endophyte patent has been estimated to be worth $3.6 billion NZD (~2.1B Euro) [37]. Conceptually, using the plant as a solar factory to produce natural biological protectants (via the symbiotic fungus) is an efficient, sustainable, way of delivering protection to broad acre pastures. For reviews on the broader exploitation of *Epichloë* endophytes for agricultural, and future perspectives, the reader is referred to recent reviews [13,35,38]. Use is still mainly limited to temperate grasses, but considerable research is aimed at extending the host range, and endophyte species used, to advance further commercial exploitation [39].

Within commercial ryegrass *Epichloë* associations, there is a dichotomy within the industry around the use of ergot alkaloids. Some groups consider ergot alkaloids to be toxic to livestock and do not support their use in current product lines. This position has arisen from fescue toxicosis observations and early trials in NZ with a high ergovaline-producing *Epichloë* Endosafe™ that did cause health issues [15,38]. Other groups take the position that it is the ‘dose that makes the poison’. For this group considerable effort has been undertaken to find products that produce low levels of ergovaline across the pasture, such that it can still confer insect resistance, but intake into the animal is low enough not to cause health issues. A review of the literature [40,41] identified theoretical non-toxic levels but admitted caveats to the research as historical work mainly used SE endophyte and often failed to note pasture alkaloid concentrations for the actual grass consumed. The review work was followed up by a study of NEA2 endophyte demonstrating that in managed pasture situations intake levels by the animal were unlikely to be above detrimental threshold levels [42]. For a review on the use of ergot alkaloid endophytes in New Zealand pastures, the reader is referred to Caradus et al. [15].

## 3. Ergot Alkaloid Profiles Produced in Epichloe Endophytes

Deployment of endophytes in grasses has primarily focused on expression of five key alkaloids—peramine, loline, lolitrem, janthitrem, and ergovaline. The myriad of associated precursors and derivatives have largely been ignored. Peramine is produced by a single enzymatic step with no intermediates and is known to be non-toxic to mammals [43]. Loline (*N*-formylloline) is produced via 3 enzymatic steps [43] and has several intermediates but like peramine is not known to be toxic to mammals [43]. Further, it is produced in endophytes primarily used in *Festuca pratensis* type grasses. The indole diterpenes lolitrems and janthitrems are toxic to mammals and have very complex pathways with many intermediates [44], some of which are also toxic. As such, the ‘simple’ determination of endpoint lolitrems (lolitrem B) and janthitrems (epoxy janthitrem I–IV) may give misleading information regarding animal health effects as intermediate and derivative levels may also have biological impact. Likewise, ergovaline is derived from a complex biochemical pathway that has among its precursors ergoline, clavines, ergoamides and ergopeptines [45,46]. The ergoline ring structure with its similarity to dopamine, serotonin and adrenaline provide ergot structures the basis to act on respective receptors as agonists or antagonists. Thus, producing a multitude of effects depending on the secondary structure. The ergopeptines ergotamine and ergovaline have similar activities in mice models [47] causing vasoconstriction, increase in blood pressure and bradycardiac properties. *Epichloe festuca var lolii* has been studied with respect to its particular ergovaline pathway [48], this analysis was for a standard toxic strain that follows the published pathway to produce a ‘standard’ level of ergovaline. Although this is often poorly determined [41]. Research in the ergot pathways has been limited to between species or across a few strain(s) within a species, which does not necessarily reflect what the profiles in particular variants might be. For example, nea2 produces a low level of ergovaline and lolitrem B [49,50]. Whilst some progress has been made in identifying the gene clusters and biochemistry present or absent in some of these strains [48,51], little work has been performed to understand differences in levels of ergot intermediates or derivatives in particular strains. For example, ergovaline is seen as a key detrimental compound on livestock health, but intermediate compounds can transfer more readily across the rumen and may in themselves cause animal health effects [52]. Finch et al. 2019 [53] studied mammalian toxicity of chanoclavine and demonstrated this to be safe, but this compound is early in the pathway. A recent study (Barenbrug NZ 2020, unpublished) using a non-lolitrem-producing strain nea3, was shown to produce high levels of paxilline and terpendole C greater than observed in SE strain and under hot dry summer conditions with ‘rank’ feed this was sufficient to cause ryegrass staggers, even though no lolitrem B was detectable. Analysis of *E. uncinata* haplotypes for loline content identified similar differences in the three forms of loline (NAL, NANL, and NFL) between different haplotypes [54] Analysis of six endophytes by Young 2013 looked at presence vs. absence of the *Eas* gene cluster and whether ergot alkaloids were produced or not [55]. The complex nature of ergot production in the Claviceps [48], between strain variation of the genes, expression profiles, and ergot bioactivity suggest that a greater level of characterisation is needed than is currently undertaken before a new endophyte strain is advanced for commercial exploitation.

## 4. Strategic Breeding Challenges with Existing Commercial Endophytes

Strategically the requirement for endophyte in a grass breeding programme throws up a conundrum. Does the breeder breed the host to fit the endophyte or breed the best ryegrass and find a compatible endophyte? The first scenario may limit the host grass germplasm to only those genotypes that fit an endophyte, e.g., work of Gagic [56]. The second strategy does not limit the host genotypes but may produce a grass that cannot sufficiently host a suitable endophyte. If several endophyte types are available, then the second strategy is possibly best but if a single endophyte is available then the first strategy is probably advisable. In NZ this has been depicted in the market through the predominant marketing of endophyte AR37, and to a much lesser extent AR1 by PGGW Seeds and Agricom brands (DLF), whilst Barenbrug have marketed NEA, NEA2, NEA4, AR1 and AR37. Most ryegrass breeding pipelines, such as ½sib family selections, and population based family selections have been developed primarily because of basic ryegrass characteristics; outcrossing, S and Z incompatibility, wind pollinated, small male and female organs situated together, and the traits of importance (biomass, persistence, heading date) [57,58]. Research efforts are slowly changing this and developments in genomic selection [58,59,60], hybrid technology [61], paternity testing [62], self-fertility [63] and biotechnology [64] will likely change the current pipelines. For now though, breeding pipelines generally revolve around ½ sib family selection [60] or between- and within-family population breeding approaches [58]. Both these systems essentially identify best families and put together phenotypically similar parent plants to make a new ‘synthetic’ cultivar. The process of performing this is very different depending on the endophyte strategy taken.

Limiting the host to fit an endophyte is a relatively easy option, and as cycles of the breeding pipeline are completed, more germplasm contains the single endophyte, and subsequent crossing and selection becomes a simple process. Introgression of ecotypes or competitor germplasm may require the elimination of any incumbent endophyte, a relatively simple heat treating of seed and testing of seedlings [65]. Then crossing with the desired endophyte-containing mother line and harvesting from these mother plants will provide introgressed offspring with the endophyte. As this material is further backcrossed checks are required to determine the stability and transmission of the endophyte. Even crosses between two lines that do contain the desired endophyte may create a host with characteristics less conducive to stability and transmission, so testing is always required. Determining compatibility in such a system, using AR37, has been made easier by the development of a genomic estimated breeding value (GEBV) tool for host acceptance of an endophyte [56]. Whilst this is a great advance, care is still required as transmission and stability within a host is not all that needs to be considered. Spatial and temporal growth, and alkaloid profile knowledge is also required, along with potential detrimental effects on the host, e.g., reduced growth. Even with a GEBV for symbiotic potential the strategic decision to use a single endophyte may limit host germplasm, such that some plant characteristics are ultimately compromised.

Breeding the best host and then finding a suitable endophyte has other challenges but does not limit the host germplasm so plant potential is not compromised. As many breeding lines and ecotypes already contain an endophyte, it is first necessary to understand what endophyte is contained and what this contributes, or detracts, to the plant phenotype. Initially, this was problematical as many endophytes were unknown and testing was a costly, skilled exercise. A small set of simple sequence repeat (SSR) were available to detect endophytes [66] with a cost ~23 Euro per test. Therefore, breeders often worked with limited knowledge, using immunological tests for endophyte presence or absence, and only confirmed type when necessary. Breeding a high-performing synthetic ryegrass population would often occur and then a subset of plants would be tested to determine endophyte status. Following this, individual plants could be chosen to constrain endophyte status. Historically at least a couple of early commercial endophyte strains were actually mixtures of two or three endophytes [20,30]. If the correct endophyte was not present, then a sample of seed could be heat cured of undesirable endophyte [65], and seedlings re-inoculated with the endophyte of choice [67]. Following this, further re-characterisation was required potentially putting the breeding programme back three years or more.

Such ‘blind’ breeding resulted in the release of a ryegrass cultivar ‘Trojan^NEA2^’ containing two endophytes, determined by SSR, and PVR protected as nea2 and nea6. This serendipitous mix provided an effective alkaloid profile and saw Trojan^NEA2^ become the top selling ryegrass across NZ between 2015 and 2018. Later, using a broader SSR panel, it was shown that the nea6 component was in fact two different endophyte strains [30], with similar alkaloid profiles. So Trojan^NEA2^ in fact consisted of nea2, nea6 and the variant endophyte [30] (later PVR protected as nea47).

Recently (Kompetitive allele specific primer), KASP technology [68] (see molecular testing below) has been applied to typing endophytes [69], which has enabled affordable identification of numerous endophyte strains quickly and easily. The system was developed for quality control determination and is based on sequence information that identifies a unique (single nucleotide polymorphism) SNP for a strain. The test result either concurs with the SNP under test, or identifies it as an ‘other’ strain, or returns a ‘below threshold/negative’. If identified as ‘other’, then this can be further investigated with different strain-specific KASP primers. KASP works very well when breeding material has known commercial strain(s), providing a simple yes/no test for identification. If unknown ecotypes are to be tested, it is also advisable to check with broad SSR panels or multiplex PCR panels such as those developed by Vikuk et al. [70].

The KASP test has transformed breeding the best host because it allows populations to be easily endophyte typed and so parents, F_1_ and F_2_ individuals can be combined with a knowledge of the endophyte. This also by default utilises two generations of selection pressure for endophyte transmission and survival such that new synthetics are created with some level of selected compatibility. Further, any plants within the synthetic that have the wrong endophyte, or no endophyte, can be eliminated, or used as pollen donors only. If two endophytes are required (as in Trojan^NEA2^ nea2/6 above), then harvest from equal numbers of mother plants containing nea2 or 6 can favour a balance of seed containing the respective endophytes, which KASP can confirm.

## 5. Practical Methods for *Epichloë*-Infected *L. perenne* Quality Control

Endophyte transmission is variable and endophyte viability decreases more quickly than the seed that contains it. Thus, moving from 100% infected nucleus seed to breeder’s seed, to G1 and G2 production seed results in a loss of endophyte. If this cumulative loss is >30% then the final seed will have insufficient endophyte to be sold as endophyte-containing seed (NZPBRA industry standard). Additionally, ingress of seed with standard toxic endophyte, or packaging, labelling, handling, and storage errors can all affect the endophyte type and percentage in the final product. New host–endophyte combination(s) also must be checked for alkaloid profile and ultimately effectiveness as an insect deterrent, and impact on animal wellbeing. Practically, this means testing for endophyte is required at all stages of the process. Such QC testing must be simple, robust, cost effective, i.e., fit for purpose. The following section provides a brief outline of common protocols used, including potential caveats, and outlines how they are used in each stage of the seed production process. They are not necessarily the latest, or all, research methods but primarily robust and cost effective.

### 5.1. Endophyte Detection—Microscopy

Tillers—Briefly, a ~1 cm base section of tiller is secured to a microscope slide using glue tape and the outer leaf blade unrolled so that it is flat against the slide. These leaf blade sections are then stained using a drop of 2% analine blue and left for ~30 min before being examined under a compound microscope (×40 magnification). Endophyte hyphae stain blue. For a more detailed description, the reader is referred to Bacon and White [71].

Seed—Briefly, ~250 seeds are placed in a test tube and 15.4 M nitric acid added to double the volume of the seeds. This is then heated at 60 °C for approximately 15 min and then the seeds are rinsed under tap water. Individual seeds are then dissected to leave the caryopsis which is stained as for the tillers above. For more detail, refer to [72].

Caveats—Endophyte is obvious growing alongside the cells of the plant. However, the strain of endophyte cannot be determined and even different endophyte species—for example, *E. occultans, E. uncinata,* or *E. typhina* can easily be mistaken for each other. Low levels of infection may go undetected and, within seeds, it should not be assumed that the endophyte is alive.

### 5.2. Endophyte Detection—Immunoblot

Briefly, tillers, cut at the base of the plant, are blotted by pressing sap from the freshly cut base onto a nitrocellulose membrane. Once blotted, the membrane is incubated with blocking solution, rinsed, and then incubated with a primary antibody. Following this, the blot is rinsed with blocking and incubated with a secondary antibody. This is rinsed off and a final chromogen mix added to bind to the secondary antibody. Blots containing endophyte protein stain according to the chromogen used. For detailed protocol, refer to [73,74], although most companies have their own custom testing regimes, a kit can be purchased from (Agrinostics, Watkinsville, GA, USA; cat. #ENDO797-3).

Caveats—Immunoblots are not strain specific and can react to different *Epichloë* species. A recent in-house comparison of seed immunoblots with microscopy revealed a small percentage of samples (~2%) were positive on the microscope and negative on the blot or vice versa (Barenbrug NZ, unpublished). Immunoblots are also based on threshold levels of detection and tiller tests use standard 6-week-old tillers, younger tillers can be used but the negative results are less reliable. Additionally, late season tillers with little sap can be difficult to blot, all of which results in semi-quantitative/qualitative data.

### 5.3. Endophyte Detection—Molecular Testing

**Figure 1:**
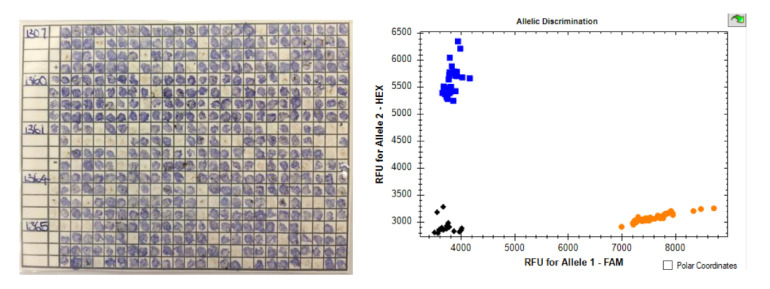
**Left**: typical immunoblot of tiller sap. Blue staining represents presence of endophyte through the detection of immunoblot antibodies bound to endophyte protein motifs. Clear squares represent ryegrass tillers without endophyte. **Right**: typical KASP assay result—in this case, assaying NEA4. Blue squares represent nea2, orange ‘other’ (in this case nea3) and black represent below threshold (negative). (Images courtesy of Amanda James.)

Molecular analysis of plant endophyte status was developed with SSRs [75]. This was rapidly developed into a fingerprinting system for endophyte types based on flanking variation around a microsatellite tandem repeat locus B [66] and soon became a routine test in NZ supplied by AgResearch. Similar systems were developed in the USA for fescue endophytes [76]. Testing requires isolation of good quality DNA from tiller or seed samples, usually via freeze dried or fresh samples, followed by multiplex assays using 3 to 5 primer sets to discerning B allele loci. Assays require electrophoretic separation of fragment sizes. Recently, using more complete sequence analysis of different endophyte strains, a catalogue of SNPs has been developed to enable the simple discrimination of known endophytes through KASP [69], this relies on fluorescent (HEX or FAM) primers competing for a binding site, which matches or mismatches the SNP in question. This process can use crudely isolated DNA and provides an immediate digital result (within 2 h, see Figure 1) via real-time PCR, reducing costs and making it suitable for in-house QC testing.

Caveats—Molecular testing requires quality assessment and threshold setting of the data. Is a negative truly negative, or just below the threshold of detection? This is particularly true if performing bulk analysis, although digital droplet PCR and or advancements in next-generation sequencing (NGS) techniques may improve this in the future. In addition, both SSR and KASP do not eliminate the risk that a new but related endophyte is wrongly detected. Using NGS techniques, it is becoming increasingly possible to identify small genetic differences even between identical twins [77], thus molecular differences depend in part on how hard you look for them.

### 5.4. Alkaloid Profiles

Testing in grass breeding situations is usually limited to analysis of leaf material, traditionally, field, or pot grown, material is usually cut to ground level. Samples are placed on ice, then frozen, freeze dried, and ground to <1 mm. This material is then analysed, via HPLC and LC–MS techniques [78,79,80]. Recently, a higher-throughput system has been developed for ergovaline, lolitrem B and peramine by Agriculture Victoria, Australia [81]. The authors investigated methanol extraction protocols, multiple separate extractions v two extractions combined and concentrated. Matrix effects and recovery efficiency was also investigated. Analysis was undertaken across three analytical platforms, two LC–MS systems, the QE MS and the QQQ MS and ergovaline was also analysed using an established HPLC-FLD method. The epoxy-janthitrem alkaloids have been very difficult to analyse due to instability issues. This has recently been overcome [82] but it is still a complex HPLC protocol requiring acetone extraction in the dark to prevent degradation. For full structural elucidation, nuclear magnetic resonance (NMR) assignments for the four epoxyjanthitrem (I–IV) compounds, and a new epoxyjanthitriol were required. The instability, extraction requirements and specific techniques for detailed analysis highlights the importance of understanding sample preparation right through to a result and highlights the difficulties of comparing data across platforms and between laboratories.

Caveats—Alkaloid analysis requires skilled biochemical knowledge, expensive equipment, and accurate sample preparation. Endophyte alkaloid profiles in a plant are difficult to sample measure and interpret and previous testing methodology and analysis has often raised more questions than it has answered [41]. Cross-laboratory validations are recommended but rarely performed due to costs and some standards such as epoxy-janthitrems are not readily available. Eady et al. [42] compared Australian and New Zealand laboratories for ergovaline and obtained a 95.5% correlation. Although other simpler techniques to detect alkaloids are being investigated such as NIR [83], for now, well-equipped laboratories with specific skills are needed for accurate alkaloid detection. These capabilities are expensive and usually result in essential analysis only being performed. For a semi-quantitative technique that can be used in house, it is possible to quantify ergot alkaloids with ELISA kits (agrinostics.com/shop/), but nothing similar is available for other alkaloids. Most of the reported alkaloid sample testing in ryegrass states ‘cut to ground level’, but even this depends upon interpretation of ground level and may vary by several cm’s (Author’s personal observation). Combine this with poor characterisation of the height of the sample, and the knowledge that alkaloid temporal or spatial spread throughout the plant also varies with host, strain and environment, and it becomes almost impossible to conclude anything beyond the individual data set [40,41]. Klotz and Nicol [40] and Nicol and Klotz [41] highlighted this in a review on animal effects caused by ergovaline in ryegrass and concluded that actual knowledge of alkaloid levels in the grass consumed was a much more relevant measure for animal health studies. It seems that the original purpose of alkaloid testing was to understand levels in plants, not levels ingested by grazing animals. Many animal health studies followed the ‘cut to ground level’ methodology, causing a reduced ability to deduce meaningful knowledge from, or compare, the results [41]. Since that review Eady et al. [42] estimated animal intake levels against the threshold level deduced by Nicol and Klotz [41]. The data would seem to agree with the threshold. However, more research is required to fully understand the impact on health of different alkaloid levels ingested through ‘natural’ grazing conditions.

### 5.5. Livestock Safety Testing

In New Zealand, evaluating novel endophyte ryegrass combinations for animal safety is undertaken voluntarily under agreed industry protocols developed by the Endophyte Technical Committee (ETC), which is part of the New Zealand Plant Breeding Research Association (https://www.nzpbra.org/ accessed on: 11 February 2021). The guidelines set out requirements for trialing including ethical approval and include scores for ryegrass staggers, heat stress, dags, and liveweight gain. Pasture under test must contain >85% viable endophyte, and chemical profiles to ground level determined. For a trial to be valid, a negative health effect must be observed in SE plots. Data are presented to the ETC and, if approved, a star rating for risk of ryegrass staggers and a comment on animal performance is assigned. Each year, industry rated tables are published to allow comparison of the animal safety parameters (https://www.nzpbra.org/ accessed on: 11 February 2021).

Caveats—Animal trialing is difficult and expensive to undertake, and such trials are under increasing pressure regarding animal welfare concerns. Data from the trials are of limited use for several reasons: (1) chemical profiles to ground level of the plant provide little knowledge as to what the actual alkaloid intake by the animal is; (2) environmental conditions vary year on year and influence alkaloid production, with trials often only run for a 4–8 week period within one year; (3) sheep genetics is not controlled and considerable variation is known to exist in regard to alkaloid tolerance; (4) the range between a valid positive control, ~1.1 ppm [84], and an extreme positive control, > 4 ppm [85], for lolitrem B alkaloid level is large; and (5) large differences in pasture quality may exist. This makes comparisons between trials difficult and may underestimate effects by up to 3.5-fold if conditions are such that only the minimum 1.1 ppm threshold is achieved. Thus, the result is open to manipulation by a skilled trial manager and the true potential of an endophyte to cause harm may be missed. The grazing practices of the trial, i.e., ‘worst-case scenario’, do not represent typical on-farm practice management and so the relevance of the test is also now under question. In addition, presence of other fungi such as *Claviceps purpurea,* or animal diseases may also cause animal fitness issues (though these should be countered by the control plots).

It may be possible with the extensive data set held by the ETC, other published material and on-going experiments, to calculate a relationship between alkaloid level and animal effect. This would allow trial material to be grown under much more defined conditions (e.g., recommended good management practice, vs. extreme, defined hot summer drought conditions) and animal alkaloid intake levels modelled by chemical analysis of the grass profiles. Such an approach would reduce environmental variables and eliminate or reduce the need for animal trials. This concept is not new, as Nicol and Klotz [41] calculated such an effect using published ergovaline data. However, for industry application, more comprehensive models are required.

### 5.6. Insect Testing

As with the animal testing, insect testing in NZ is via industry-agreed protocols developed by the ETC. Six key pest insects affecting NZ pastures have agreed testing protocols. Testing is customised for each insect but generally involves pot and/or field trials against a susceptible (no endophyte) and control, typically SE plants. Insects under question include Argentine stem weevil (*Listronotus bonariensis*), black beetle (*Heteronychus arator*), grass grub (*Costelytra zealandica*), pasture mealybug (*Balanococcus poae*), porina (*Wiseana spp*.), and root aphid (*Aploneura lentisci*). Other insect pests have also been investigated, and ryegrass *Epichloë* alone have been demonstrated to have activity against over 20 insect species [86]. As needs arise, or commercial advantage is sought, it is likely that additional protocols will be added to the current list of six. Each year, data are submitted to the ETC and industry-approved star rating tables for endophyte insect control are published by the NZPBRA (https://www.nzpbra.org/ accessed on: 11 February 2021).

Caveats—As with livestock testing, insect testing is difficult, with many variables requiring control. Health and life stage of the insect, genetics of the populations, health and maturity stage of the plant, and environmental conditions all impact upon the response. These can cause variability between trials in pots, and in-field trials where infestation, or not, is heavily influenced by geography and season. Trials require entomological expertise, and like many livestock experiments are often contracted out to universities or research organisations at considerable expense.

## 6. Use of Quality Control Methods within a Ryegrass Seed Company

### 6.1. Breeding

Once a strategy of how to breed has been decided (see Section 4), then a further strategy on what to test and when must be established. Determining the order of importance of endophyte traits and when and where to test for them through the breeding pipeline is a perplexing task. Any new endophyte discovered, serendipitously, or via screening of seed banks or collections is usually initially phylogenetically identified through SSR, KASP, or NGS based molecular analysis. The new endophyte is then assessed in its natural host and an alkaloid profile deduced (although this is rarely spatially and temporally studied at these early stages). If desirable, transfer to elite host germplasm follows then a repeated round of alkaloid profiling (1–2 years) is undertaken. Ability of the endophyte to be inoculated into a broad range of host germplasm is also desirable but, this is a resource dependent process, as most inoculations are only successful at a low frequency (~4–20%). Stability and transmission tend to be studied in parallel to field evaluations that also investigate the effect of the endophyte on host architecture and performance. Research groups such as those at the Noble Research Institute (www.noble.org accessed on: 11 February 2021), AgResearch (www.agresearch.co.nz accessed on: 11 February 2021), and Agribio (www.agribio.com.au accessed on: 11 February 2021) have research teams dedicated to this process and usually work collaboratively with seed companies.

The success rate of this discovery process has declined with time as it becomes increasingly unlikely to find a novel and useful profile, within the finite species/strains available [87]. Research, e.g., Kaur et al. [88] has looked at many hundreds of *Epichloë* endophytes, of these, few have proceeded beyond their initial screening. The New Zealand PVR database lists 34 endophytes (www.iponz.govt.nz accessed on: 11 February 2021), of these, 3 have expired, 4 have been withdraw, 24 granted, and only 3 are currently filed. Application dates and numbers suggest (given discovery is some time before application) a peak discovery before 2012–2014 for ryegrass endophytes (Table 2).

Inoculation into elite germplasm requires at least 40 individual ryegrass genotypes to be successfully inoculated, otherwise the allele frequency of the host may shift significantly from the original elite germplasm [89]. An alternative way if inoculation is difficult is to inoculate a few plants and then cross onto them with many different elite pollen donor genotypes. If inoculation is not possible, then backcrossing onto the original host, acting as the mother, can also be undertaken. This method may help by introgression of host genes, required for endophyte stability, into the elite germplasm, but requires at least four rounds of backcrossing to get a genotype similar to the original elite germplasm.

In mature breeding pipelines regardless of strategy, existing elite germplasm already contains known functional endophyte(s). With such material the process is easier, and by selective harvesting from mother plants endophyte status can be controlled and new synthetics created. Whatever method is used, each stage of the process needs to be monitored by tiller immunoblots to confirm inoculation, and viable transmission. Microscopy or immunoblots of seeds for presence of the endophyte in the seed is also required. Subsamples of plants, or seed need to be checked by molecular analysis to confirm strain and detect any contamination. Information on transmission, stability and effect on host phenotype is gathered, as resource allows, during the breeding of the new ‘synthetic’ line. Failure of any of these characteristics can prevent the new endophyte/host symbiont from progressing.

### 6.2. Agronomy

Once a new ryegrass synthetic line x endophyte strain(s) combination has been chosen for advancement, it requires agronomic assessment. Initially, confirmation of the alkaloid profile, temporally and spatially, is undertaken, along with nationwide trials to understand the host performance. If chosen for advancement, then animal and insect trials, following ETC testing protocols, can be undertaken. For all these trials, seed batches and tiller samples again need to be tested for endophyte presence and subtested for endophyte strain to ensure QC through the process.

### 6.3. Production

Seedlings of the new line (putative cultivar) are tested for endophyte using immunoblots, and a subset for molecular profiling, any negatives are removed, and seed is harvested from 100% infected plants. This is then multiplied up by seed production specialists within the companies. Some marketed endophytes are a mixture of two different endophytes, e.g., NEA2. Initially, this was serendipitously multiplied up as a mixture but now at each stage of multiplication the ratio of the strains is monitored through molecular testing. Alternatively mixtures can be made by combining individual host–endophyte combinations [90] at various stages of the multiplication process. For single strains, testing is simpler but still required to ensure that no contamination or mislabelling has occurred. Additionally, because endophyte transmission is rarely 100%, endophyte seed percentage, and tiller viability assessments are required at each stage of multiplication from breeders, to pre-basic, to certified seed generation 1 and 2. The final seed for sale requires an endophyte viability of above 70%, otherwise it has to be sold as a low-endophyte product. Despite the importance of transmission and viability, it still varies through management and climatic conditions. Research in this area [35,74,91,92,93], and considerable in-house research, has made some advancement but endophyte status in production crops is still a major production risk. In addition, crop treatments such as pesticides (especially fungicides) have to be evaluated for potential negative effects on endophyte status [92]. Loss of endophyte status can require the production manager to rework the cultivar starting with a new 100% infected batch of nucleus plants. For new host–endophyte combinations, specific testing of fungicide applications, plant growth regulators, and seed treatments, on the transmission and viability of the endophyte may be necessary.

### 6.4. Storage and Sale

Endophyte viability in the seed declines quicker than the host seed does [94,95,96,97] and this rate varies depending upon the relationship and the storage environment [98]. Storage at low temperature and humidity is required to maintain endophyte viability and insure delivery of good quality product to the farmer [99]. Most seed companies involved in endophyte have strict harvest and storage conditions to quickly cool the seed and maintain low humidity, required for endophyte longevity. This includes strict timelines from harvest to cool storage. Within the NZ industry, freshly harvest seed is given a 6 month grace period, whereby machine dressed endophyte level (seed squash or immune test) is accepted for viability. Following that seed lots must be tested every 6 months, usually via a 6 week tiller grow-out test to ensure viability of the endophyte. This is a logistical problem as failure to maintain testing can see lines requested for immediate sale having to wait 6 weeks for a test result. Farmers may go elsewhere for product under such a scenario. Thus, efforts to reduce the time required to perform seed viability tests are important (see Section 5.2 above). Sale to farmers is usually via a third-party store and ‘just in time’ delivery to these stores reduces the likelihood of poor storage between leaving the production company and sale to the farmer. Advice on on-farm seed storage is given, but there is little monitoring, and once the grass is sown in the paddock little or no monitoring is undertaken. Occasionally, if a poor paddock is produced checks on endophyte status can be made using molecular profiling. This has resulted in the identification of wrong product, or products seemingly mixed with other products (Author, unpublished). Buying from a reputable dealer can help avoid this. Using endophyte status as a QC or product marker is another useful attribute that endophyte can confer to a ryegrass product.

## 7. Opportunities and Risks for the Future

### 7.1. Host Breeding

As mentioned above, ryegrass breeding methods are improving, new phenotyping [64] and genotyping techniques [57] are starting to deliver cost effective genomic selection [58,60,100] and these are being applied to improve endophyte stability and transmission [56]. Addition of hybrid breeding [61], self-fertility [101], along with gene editing technologies [102] will likely reduce variation between genotypes within a host germplasm (cultivar or F1 hybrid). This reduced G × G variation would be expected to result in a more consistent endophyte profile within the product. Still factors affecting the symbioses are many and complex. As an example, Rinklake et al. [14] recently proposed that the process of vernalisation maybe an important transmission factor. Endophyte has a different cardinal temperature to ryegrass and the range of cold required for ryegrass vernalisation may affect endophyte growth and triggers for establishment of reproductive tillers differentially. This could lead to reduced endophyte entering the newly formed tiller, where, through intercalary growth [103], it eventually enters the developing infloresences [103]. Reduced initial colonisation of the shoot apical meristem may therefore reduce the number of infloresences infected. Knowing what traits are important in stability and transmission, are key to future breeding efforts. In a completely different breeding approach, Spangenberg (European Patent Office EP2521442A1) has proposed growing and selecting populations of ryegrass containing multiple different endophyte strains in vivo and simply selecting for the best plants to create a host population containing a plurality of endophytes [104].

### 7.2. Endophyte Discovery and Manipulation

Discovery and exploitation have become increasingly sophisticated with the use of NGS [12] and high-throughput biochemical analysis techniques [81]. There is no doubt that discovery programmes will continue but as stated this will require smarter or greater efforts to discover new, useful, endophytes. Increasing ease of certain techniques has widened the scope for who can look, and existing collections may still hold interesting endophytes. A recent NZ PVR for CM142 demonstrates this, as it was found in an existing collection from the Margot Forde Germplasm Centre [105]. Use of molecular QC analysis is now routine for many companies and provides opportunities to detect ‘unknown’ endophytes. This expands discovery capability that was previously limited to a few research laboratories around the world. Aside from discovery it is possible to screen endophyte/host cultivar populations and identify individuals that express different levels of alkaloid. This can be used to select for a particular profile such as lower ergovaline levels. Ryegrass cultivar Reward containing Endo5 endophyte is an example of such a product being a subselection of AR5, itself a subselection of Endosafe™, with evidence for this shown by their interconnectedness in the paper by Thom et al. [20]).

Genetic modification (GM) and/or gene editing techniques can be used to manipulate the asexual endophyte. With no sexual recombination breeders are reliant on somatic mutations within the endophyte. Evolutionarily hybridisations have arisen but attempts to achieve this in vitro have been unsuccessful to date and use of mutagens invokes Muller’s ratchet making accumulation of deleterious mutations far more likely than a specific beneficial one [106]. GM research has been demonstrated in *Epichloë* for a considerable time [107,108] and some site-specific mutagenesis using NHEJ [109], and recently the use of CRISPR/Cas9 gene editing has been demonstrated [110]. Due to regulatory hurdles, GM research is limited to laboratory research activities, but gene editing, having been deregulated in many countries, offers up some unique opportunities. Precise editing of toxin profiles is an obvious target and repair of a perA gene within a LPTG-3 janthitrem-producing endophyte, as currently achieved through GM [107], a potential candidate. If GM becomes acceptable, then adding more complex multigene alkaloid pathways to make a customised profiles might be possible.

### 7.3. Deployment Systems

Beyond genetic manipulation of endophyte and host, through conventional breeding, mutagenesis or biotechnology, there is also the opportunity to improve endophyte functionality in the pasture through creative deployment systems. One method is simply to dilute the intake by the animal through growing multiple species within the pasture. This is currently a topical approach as regenerative agricultural (RA) proponents advocate multiple species pastures. However, NZ soils already have high, stable carbon reserves [111], thus negating a major proposed benefit of RA. Additionally, ryegrass has an annual biomass, ME, and intake preference advantage in much of NZ [112] so RA would likely dilute production gains achieved by current proprietary ryegrass/endophyte pastures. If diverse species and a more ‘natural’ habitat is the goal of RA, then perhaps sustainable intensification and set aside land with native species maybe a better approach. Diluting alkaloid levels without losing production could be overcome by blending with a non-endophyte-containing ryegrass, and such products are currently sold as LE (low-endophyte) ryegrass in NZ. However, under high insect pressure without-endophyte grass might be preferentially predated leaving the high alkaloid ryegrass. In reality, it seems that if this occurs, then weeds fill the space and maintain the alkaloid dilution [42]. However, weeds are defined by their poor feed value or growth, so animal performance is again potentially is lost. A second and more sophisticated strategy would be to design novel endophyte combinations within the sward. NEA2 contains nea2 strain, a low ergovaline producer and nea6 strain, a moderate ergovaline producer, resulting in a sward with an ergovaline level sufficient to confer good insect protection but low enough to be unlikely to cause animal health issues under normal grazing conditions [42]. Alternatively, a farmer can purchase two different endophyte-containing cultivars—one with ergovaline and peramine and another producing janthitrem—this could provide a broad spectrum of excellent insect protection properties and reduce the individual alkaloid levels within the pasture. As janthitrem and ergovaline have different effects on livestock, neurological (janthitrem) vs. physiological (ergovaline), the dilution helps prevent either reaching a threshold that will cause animal health issues. Designer mixtures within the best-performing cultivar has been proposed [90] and offers the additional opportunity of maintaining optimal pasture yield. Currently in NZ nitrogen leaching issues are encouraging farmers to reduce synthetic fertiliser application and increase clover and herb (plantain or chicory) percentage within the pasture. This science-based approach results in a diverse pasture blend (ryegrass, clover, herb, endophyte, rhizobia) with effective alkaloid levels, whilst maintaining a productive and sustainable pasture.

### 7.4. Risks

The success of ergovaline- and non-ergovaline-producing endophytes in New Zealand ryegrass is well documented and without endophytes insect pests can devastate pastures, especially in the North Island. However, this situation is unique, and uptake and deployment in Australia, South America, Africa, Europe and North America has been much less. Fescue toxicosis, ryegrass staggers and ergot poisoning are key contributors behind this restraint and represent real issues with historically severe outbreaks reported in many countries [113] that have led to numerous animal deaths. In addition, many other regions of the world are not suited, or reliant, on the almost exclusive temperate ryegrass growth that is practiced in New Zealand. Different grass species require different endophytes, different insects require different alkaloid deterrents, greater reliance on other feed detracts from the value of the endophyte, annual production over perennial, and other factors all change the commercial equation that is made in estimating the cost to benefit ratio, the risk, of using endophyte. Climate change will alter insect pests within regions of the globe and what pasture species are grown within specific regions. Such changes are both risk and opportunity for endophyte-based alkaloid deployment but given the long lead in time (6 to 16 years) to incorporate new endophytes into ryegrass cultivars, these decisions also represent a risk to the breeding companies concerned.

## 8. Conclusions

Plant breeding of ryegrass would be much simpler without endophytes. Most breeding data management software have issues coping with a maternally inherited trait that can be lost, added, or interchanged with a different ‘trait’. For the breeder, following the trait, and all the QC required around this, is an enormous drain on resources. Having to re-test the performance of a novel host germplasm with a new endophyte can take three years from re-inoculation. An inability to routinely produce commercial quantities of seed with sufficient (>70%) viable endophyte can see a huge loss in margin for that crop, and disrupt supply leading to many marketing issues. Likewise, loss of endophyte viability in storage is a logistical challenge requiring constant re-evaluation and re-prioritising of stock for sale. Contamination with wild-type endophyte, or another proprietary endophyte, for example through growing on contaminated land, can see a crop production ruined, and if unknowingly on-sold cost millions in reparation costs. This recently happened with an Australian tall fescue cultivar Barnaby [114]. Sale of the wrong endophyte for consumption by deer or horses, which are more sensitive to alkaloids, can also result in large lawsuits or reparation costs.

Despite the catalogue of negatives, *Epichloë* endophytes have stood the test of time and proved to be a vital component of NZ pasture systems. The obvious natural, sustainable protection that *Epichloë* alkaloids offer to pasture is too valuable to ignore. Farming systems continue to come under ever increasing pressure to ‘be natural’ and many synthetic pesticides are being removed from the market. This provides unique opportunities that will no doubt see the use of endophytes within and beyond pasture systems expand in the future. In addition, the new knowledge of host–endophyte associations along with better breeding capability for the associations will see improved production and deployment. Finally, the deregulation (in many parts of the world) and use of gene editing, and other biotechnologies, bring an exciting capability to customise alkaloid profiles and or other attributes of the *Epichloë* endophyte to potentially provide new sustainable solutions for the agriculture industry.

## Figures and Tables

**Table 1:** Lists commercial endophytes, their botanical name, principal host, secondary host in brackets, the PVR approval date and the alkaloid profile, P = peramine, L = lolines, E = ergovaline, Lol = lolitrem, and J = epoxy-janthitrems. * Only basic endpoint compounds identified as data from PVR reports and manuscripts often fail to detail all compounds tested, sampling protocols, temporal and spatial sampling differences, thus making relativities difficult to assess.

Endophyte	Botanical Name	Grass Host	PVR Reg.	Alkaloid Profile *
AR1	Epichloe festucae Leuchtm., Schardl and M. R. Siegel	Perennial Ryegrass	23 April 1996 (expired)	P
AR501	Epichloe coenophiala C.W. Bacon and Schardl	Tall Fescue (Perennial Ryegrass)	23 April 1996 (expired)	P, L
AR542 (MaxP)	Epichloe coenophiala C.W. Bacon and Schardl	Tall Fescue	1 February 1999 (expired)	P. L
UNC1	Epichloe uncinata Leuchtm. and Schardl	Meadow Fescue	14 October 2008	L
nea 2	Epichloe festucae Leuchtm., Schardl and M. R. Siegel	Perennial Ryegrass	25 July 2008	P, E, Lol
AR37	Epichloe festucae Leuchtm., Schardl and M. R. Siegel	Perennial Ryegrass	25 July 2008	J
Happe	Epichloe (Fr.) Tul. and C. Tul. (Epichloë siegelii)	Meadow Fescue (Perennial Ryegrass, Festulolium)	23 June 2010	L
nea 3	Epichloe festucae Leuchtm., Schardl and M. R. Siegel	Perennial Ryegrass	30 June 2009	E, P
U2	Epichloe uncinata Leuchtm. and Schardl	Meadow Fescue	25 July 2008	L
nea 6	Epichloe festucae Leuchtm., Schardl and M. R. Siegel	Perennial Ryegrass	25 July 2008	E, P
AR584 (MaxQ)	Epichloe coenophiala C.W. Bacon and Schardl	Tall Fescue	25 July 2008	L, P
AR95 (Avanex^®^)	Epichloe festucae Leuchtm., Schardl and M. R. Siegel	Perennial Ryegrass	28 August 2014	E
PTK647	Epichloe coenophiala C.W. Bacon and Schardl	Tall Fescue	18 August 2014	E, L
AR601 (Avanex^®^)	Epichloe coenophiala C.W. Bacon and Schardl	Tall Fescue	12 May 2010	E, L
AR604	Epichloe coenophiala C.W. Bacon and Schardl	Tall Fescue	12 May 2010	E, L
U12	Epichloe uncinata Leuchtm. and Schardl	Meadow Fescue	28 August 2014	L
AR1006	Epichloe uncinata Leuchtm. and Schardl	Meadow Fescue	21 April 2015	L
E815	Epichloe festucae Leuchtm., Schardl and M. R. Siegel	Perennial Ryegrass	27 August 2014	P, E, Lol
nea10	Epichloe festucae Leuchtm., Schardl and M. R. Siegel	Perennial Ryegrass	29 August 2014	E, P
nea11	Epichloe (Fr.) Tul. and C. Tul.	Perennial Ryegrass	13 August 2014	E, P
nea21	Epichloe (Fr.) Tul. and C. Tul.	Meadow Fescue (Perennial Ryegrass)	29 August 2014	L, P
nea23	Epichloe (Fr.) Tul. and C. Tul.	Meadow Fescue (Perennial Ryegrass)	29 August 2014	L, P
U13	Epichloe uncinata Leuchtm. and Schardl	Meadow Fescue	2 September 2016	L
AR1017	Epichloe uncinata Leuchtm. and Schardl	Meadow Fescue	5 October 2016	L
CM142	Epichloe festucae Leuchtm., Schardl and M. R. Siegel	Perennial Ryegrass	17 January 2019	J
nea47	Epichloe festucae Leuchtm., Schardl and M. R. Siegel	Perennial Ryegrass	10 July 2019	E, P

**Table 2:** *Epichloë* PVR applications granted in NZ 1997 to 2020.

Year	1997–1999	2000–2002	2003–2005	2006–2008	2009–2011	2012–2014	2015–2017	2018–2020
*Epichloë* PVR applications	1	2	2	5	4	10	5	2

## Data Availability

Not applicable.

## References

[1] Hhe V.Z., Herrero M., Van Hal O., Röös E., Muller M., Garnett T., Gerber P., Schader C., De Boer L. (2018). Defining a Land Boundary for Sustainable Livestock Consumption. Glob. Chang. Biol..

[2] Lorenz H., Reinsch T., Hess S., Taube F. (2019). Is Low-Input Dairy Farming More Climate Friendly? A Meta-Analysis of the Carbon Footprints of Different Production Systems. J. Clean. Prod..

[3] Chobtang J., Ledgard S., Mclaren S., Donaghy D.J. (2017). Life Cycle Environmental Impacts of High and Low Intensification Pasture-Based Milk Production Systems: A Case Study of the Waikato Region, New Zealand. J. Clean. Prod..

[4] Case B., Ryan C. (2020). An Analysis of Carbon Stocks and Net Carbon Position for New Zealand Sheep and Beef Farmland. https://beeflambnz.com/sites/default/files/news-docs/BL_Carbon_report_for_review_final_submit.pdf.

[5] Stewart A. (2006). Genetic Origins of Perennial Ryegrass (Lolium Perenne) for New Zealand Pastures. Adv. Plant Breed..

[6] Monks D.P., Sadat Asilan K., Moot D.J. (2009). Cardinal Temperatures and Thermal Time Requirements for Germination of Annual and Perennial Temperate Pasture Species. Agron. N. Z..

[7] Crush J.R., Rowarth J.S. (2007). The Role of C_4_ Grasses in New Zealand Pastoral Systems. N. Z. J. Agric. Res..

[8] Rowarth J.S., Pennell C.G., Fraser T.J., Baird D.B. (1996). Pasture Response to Fertiliser Inputs under Dairy Grazing. Proc. N. Z. Grassl. Assoc..

[9] Schardl C.L., Leuchtmann A., Spiering M.J. (2004). Symbioses of Grasses with Seedborne Fungal Endophytes. Annu. Rev. Plant Biol..

[10] Schardl C.L., Collett M.A., Watt D., Scott D.B. (1994). Origin of a Fungal Symbiont of Perennial Ryegrass by Interspecific Hybridization of a Mutualist with the Ryegrass Choke Pathogen. Genetics.

[11] Saikkonen K., Young C.A., Helander M., Schardl C.L. (2016). Endophytic Epichloë Species and Their Grass Hosts: From Evolution to Applications. Plant Mol. Biol..

[12] Hettiarachchige I.K., Ekanayake P.N., Mann R.C., Guthridge K.M., Sawbridge T.I., Spangenberg G.C., Forster J.W. (2015). Phylogenomics of Asexual Epichloë Fungal Endophytes Forming Associations with Perennial Ryegrass. BMC Evol. Biol..

[13] Johnson L.J., Voisey C.R., Faville M.J., Moon C.D., Simpson W.R., Johnson R.D., Stewart A.V., Caradus J.R., Hume D.E. Advances and Perspectives in Breeding for Improved Grass-Endophyte Associations. Proceedings of the “Improving Sown Grasslands through Breeding and Management” Joint Symposium EFG/Eucarpia.

[14] Rinklake I., James A., Brownfield L., MacKnight R., Eady C. (2020). Relative Epichloë Endophyte Fungal Biomass in Ryegrass Tillers Grown Pre and Post Vernalization. N. Z. Agron. Soc. J..

[15] Caradus J.R., Card S.D., Finch S.C., Hume D.E., Johnson L.J., Mace W.J., Popay A.J. (2020). Ergot Alkaloids in New Zealand Pastures and Their Impact. N. Z. J. Agric. Res..

[16] Neill J.C. (1940). The Endophyte of Rye-Grass (Lolium Perenne). N. Z. J. Sci. Technol. Sect. A.

[17] Bacon C.W., Porter J.K., Robbins J.D., Luttrell E.S. (1977). Epichloë Typhina from Toxic Tall Fescue Grasses. Appl. Environ. Microbiol..

[18] Fletcher L.R., Harvey I.C. (1981). An Association of a Lolium Endophyte with Ryegrass Staggers. N. Z. Vet. J..

[19] Leuchtmann A., Bacon C.W., Schardl C.L., White J.F., Tadych M. (2014). Nomenclatural Realignment of *Neotyphodium* Species with Genus Epichloë. Mycologia.

[20] Thom E.R., Popay A.J., Hume D.E., Fletcher L.R. (2012). Evaluating the Performance of Endophytes in Farm Systems to Improve Farmer Outcomes—A Review. Crop Pasture Sci..

[21] Sewell J.C. (2015). Recurrent Selection in Perennial Ryegrass (Lolium Perenne L.) for Reduced Levels of Ergovaline with Particular Emphasis on the Effect of Other Ergot Alkaloid Concentrations. Master’s Thesis.

[22] Fletcher L.R., Popay A.J., Tapper B.A. (1991). Evaluation of Several Lolitrem-Free Endophyte/Perennial Ryegrass Combinations. Proc. N. Z. Grassl. Assoc..

[23] Latch G.C.M. (1994). Influence of *Acremonium* Endophytes on Perennial Grass Improvement. N. Z. J. Agric. Res..

[24] Fletcher L.R., Sutherland B.L., Fletcher C.G. (1999). The Impact of Endophyte on the Health and Productivity of Sheep Grazing Ryegrass-Based Pastures. Grassl. Res. Pract. Ser..

[25] Popay A.J., Hume D.E., Baltus J.G., Latch G.C.M., Tapper B.A., Lyons T.B., Cooper B.M., Pennell C.G., Eerens J.P.J., Marshall S.L. (1999). Field Performance of Perennial Ryegrass (Lolium Perenne) Infected with Toxin-Free Fungal Endophytes (*Neotyphodium Spp.*). Grassl. Res. Pract. Ser..

[26] Thom E.R., Waugh C.D., Minneé E.M.K., Waghorn G.C. (2013). Effects of Novel and Wild-Type Endophytes in Perennial Ryegrass on Cow Health and Production. N. Z. Vet. J..

[27] Hume D.E., Stewart A.V., Simpson W.R., Johnson R.D. (2020). *Epichloë* Fungal Endophytes Play a Fundamental Role in New Zealand Grasslands. J. R. Soc. N. Z..

[28] Bultman T.L. (2006). Neotyphodium in Cool-Season Grasses. Crop Sci..

[29] Hume D.E., Cooper B.M., Panckhurst K.A. (2009). The Role of Endophyte in Determining the Persistence and Productivity of Ryegrass, Tall Fescue and Meadow Fescue in Northland. Proc. N. Z. Grassl. Assoc..

[30] Fletcher L., Finch S., Sutherland B., deNicolo G., Mace W., van Koten C., Hume D. (2017). The Occurrence of Ryegrass Staggers and Heat Stress in Sheep Grazing Ryegrass-Endophyte Associations with Diverse Alkaloid Profiles. N. Z. Vet. J..

[31] Finch S.C., Thom E.R., Babu J.V., Hawkes A.D., Waugh C.D. (2013). The Evaluation of Fungal Endophyte Toxin Residues in Milk. N. Z. Vet. J..

[32] Reddy P., Guthridge K., Vassiliadis S., Hemsworth J., Hettiarachchige I., Spangenberg G., Rochfort S. (2019). Tremorgenic Mycotoxins: Structure Diversity and Biological Activity. Toxins.

[33] Thom E.R., Waugh C.D., Minnee E.M.K. A New Generation Ryegrass Endophyte—The First Results from Dairy Cows Fed AR37. Proceedings of the 6th International Symposium on Fungal Endophytes of Grasses.

[34] Kitson E.R. (2017). Viability of Endophytic Fungus in Different Perennial Ryegrass (Lolium Perenne) Varieties Kept in Different Storage Conditions. Master‘s Thesis.

[35] Johnson L.J., Caradus J.R., Hodkinson T.R., Doohan F.M., Saunders M.J., Murphy B.R. (2019). The Science Required to Deliver *Epichloë* Endophytes to Commerce. Endophytes for a Growing World.

[36] Use of Grass Fungi Saving NZ Billions Agriculture Science. https://www.agresearch.co.nz/news/use-of-grass-fungi-saving-nz-billions/.

[37] Rivero M.J., Lee M.R.F., Cone J.W. (2018). The Role of Pasture in the Diet of Ruminant Livestock. In Balancing Pasture Productivity with Environmental and Animal Health Requirements.

[38] Johnson L.J., de Bonth A.C.M., Briggs L.R., Caradus J.R., Finch S.C., Fleetwood D.J., Fletcher L.R., Hume D.E., Johnson R.D., Popay A.J. (2013). The Exploitation of Epichloae Endophytes for Agricultural Benefit. Fungal Divers..

[39] Panaccione D.G., Beaulieu W.T., Cook D., Allen E. (2014). Bioactive Alkaloids in Vertically Transmitted Fungal Endophytes. Funct. Ecol..

[40] Klotz J.L., Nicol A.M. (2016). Ergovaline, an Endophytic Alkaloid. 1. Animal Physiology and Metabolism. Anim. Prod. Sci..

[41] Nicol A.M., Klotz J.L. (2016). Ergovaline, an Endophytic Alkaloid. 2. Intake and Impact on Animal Production, with Reference to New Zealand. Anim. Prod. Sci..

[42] Eady C.C., Corkran J.R., Bailey K.M., Kerr G.A., Nicol A.M. (2017). Estimation of Ergovaline Intake of Cows from Grazed Perennial Ryegrass Containing NEA2 or Standard Endophyte. J. N. Z. Grassl..

[43] Schardl C.L., Grossman R.B., Nagabhyru P., Faulkner J.R., Mallik U.P. (2007). Loline Alkaloids: Currencies of Mutualism. Phytochemistry.

[44] Ludlow E.J., Vassiliadis S., Ekanayake P.N., Hettiarachchige I.K., Reddy P., Sawbridge T.I., Rochfort S.J., Spangenberg G.C., Guthridge K.M. (2019). Analysis of the Indole Diterpene Gene Cluster for Biosynthesis of the Epoxy-Janthitrems in Epichloë Endophytes. Microorganisms.

[45] Panaccione D.G. (2005). Origins and Significance of Ergot Alkaloid Diversity in Fungi. FEMS Microbiol. Lett..

[46] Gerhards N., Neubauer L., Tudzynski P., Li S.-M. (2014). Biosynthetic Pathways of Ergot Alkaloids. Toxins.

[47] Reddy P., Hemsworth J., Guthridge K.M., Vinh A., Vassiliadis S., Ezernieks V., Spangenberg G.C., Rochfort S.J. (2020). Ergot Alkaloid Mycotoxins: Physiological Effects, Metabolism and Distribution of the Residual Toxin in Mice. Sci. Rep..

[48] Florea S., Panaccione D.G., Schardl C.L. (2017). Ergot Alkaloids of the Family Clavicipitaceae. Phytopathology.

[49] Tian P., Le T.-N., Ludlow E.J., Smith K.F., Forster J.W., Guthridge K.M., Spangenberg G.C. (2013). Characterisation of Novel Perennial Ryegrass Host–Neotyphodium Endophyte Associations. Crop Pasture Sci..

[50] Jong E.V.Z.D., Dobrowolski M.P., Sandford A., Smith K.F., Willocks M.J., Spangenberg G.C., Forster J.W. (2008). Detection and Characterisation of Novel Fungal Endophyte Genotypic Variation in Cultivars of Perennial Ryegrass (Lolium Perenne L.). Aust. J. Agric. Res..

[51] Lorenz N., Haarmann T., Pažoutová S., Jung M., Tudzynski P. (2009). The Ergot Alkaloid Gene Cluster: Functional Analyses and Evolutionary Aspects. Phytochemistry.

[52] Hill N.S., Thompson F.N., Stuedemann J.A., Rottinghaus G.W., Ju H.J., Dawe D.L., Hiatt E.E. (2001). Ergot Alkaloid Transport across Ruminant Gastric Tissues. J. Anim. Sci..

[53] Finch S.C., Munday J.S., Sprosen J.M., Bhattarai S. (2019). Toxicity Studies of Chanoclavine in Mice. Toxins.

[54] Cagnano G., Roulund N., Jensen C.S., Forte F.P., Asp T., Leuchtmann A. (2019). Large Scale Screening of Epichloë Endophytes Infecting Schedonorus Pratensis and Other Forage Grasses Reveals a Relation Between Microsatellite-Based Haplotypes and Loline Alkaloid Levels. Front. Plant Sci..

[55] Young C.A., Hume D.E., McCulley R.L. (2013). Forages and Pastures Symposium: Fungal Endophytes of Tall Fescue and Perennial Ryegrass: Pasture Friend or Foe?. J. Anim. Sci..

[56] Gagic M., Faville M.J., Zhang W., Forester N.T., Rolston M.P., Johnson R.D., Ganesh S., Koolaard J.P., Easton H.S., Hudson D. (2018). Seed Transmission of Epichloë Endophytes in Lolium Perenne Is Heavily Influenced by Host Genetics. Front. Plant Sci..

[57] Faville M.J., Ganesh S., Cao M., Jahufer M.Z.Z., Bilton T.P., Easton H.S., Ryan D.L., Trethewey J.A.K., Rolston M.P., Griffiths A.G. (2018). Predictive Ability of Genomic Selection Models in a Multi-Population Perennial Ryegrass Training Set Using Genotyping-by-Sequencing. Theor. Appl. Genet..

[58] Pembleton L.W., Inch C., Baillie R.C., Drayton M.C., Thakur P., Ogaji Y.O., Spangenberg G.C., Forster J.W., Daetwyler H.D., Cogan N.O.I. (2018). Exploitation of Data from Breeding Programs Supports Rapid Implementation of Genomic Selection for Key Agronomic Traits in Perennial Ryegrass. Theor. Appl. Genet..

[59] Faville M.J., Richardson K., Gagic M., Mace W., Sun X.Z., Harrison S., Knapp K., Jahufer M.Z.Z., Palanisamy R., Pirlo S. (2010). Genetic improvement of fibre traits in perennial ryegrass. Proc. N. Z. Grassl. Assoc..

[60] Faville M., Cao M., Schmidt J., Ryan D., Ganesh S., Jahufer M., Hong S., George R., Barrett B. (2020). Divergent Genomic Selection for Herbage Accumulation and Days-To-Heading in Perennial Ryegrass. Agronomy.

[61] Pembleton L.W., Shinozuka H., Wang J., Spangenberg G.C., Forster J.W., Cogan N.O.I. (2015). Design of an F1 Hybrid Breeding Strategy for Ryegrasses Based on Selection of Self-Incompatibility Locus-Specific Alleles. Front. Plant Sci..

[62] Riday H. (2011). Paternity Testing: A Non-Linkage Based Marker-Assisted Selection Scheme for Outbred Forage Species. Crop Sci..

[63] Do Canto J., Studer B., Frei U., Lübberstedt T. (2018). Fine Mapping a Self-Fertility Locus in Perennial Ryegrass. Theor. Appl. Genet..

[64] Ghamkhar K., Irie K., Hagedorn M., Hsiao J., Fourie J., Gebbie S., Hoyos-Villegas V., George R., Stewart A., Inch C. (2019). Real-Time, Non-Destructive and in-Field Foliage Yield and Growth Rate Measurement in Perennial Ryegrass (Lolium Perenne L.). Plant Methods.

[65] Kirkby K.A., Hume D.E., Pratley J.E., Broster J.C. Effect of Temperature on Endophyte and Plant Growth of Annual Ryegrass, Perennial Ryegrass and Tall Fescue. Proceedings of the 17th Australasian Weeds Conference, New Frontiers in New Zealand: Together We Can Beat the Weeds.

[66] Moon C.D., Tapper B.A., Scott B. (1999). Identification of Epichloë Endophytes In Planta by a Microsatellite-Based PCR Fingerprinting Assay with Automated Analysis. Appl. Environ. Microbiol..

[67] Latch G.C.M., Christensen M.J. (1985). Artificial Infection of Grasses with Endophytes. Assoc. Appl. Biol..

[68] Semagn K., Babu R., Hearne S., Olsen M. (2014). Single Nucleotide Polymorphism Genotyping Using Kompetitive Allele Specific PCR (KASP): Overview of the Technology and Its Application in Crop Improvement. Mol. Breed..

[69] Kaur J., Gutheridge K.M., Sawbridge T.I., Mann R.M., Forster J.W., Spangenberg G.C. SNP Specific Genotyping of Pasture Grass Endophytes Using KASP (Kompetitive Allele Specific PCR) Assay. Proceedings of the 9th International Symposium on Fungal Endophytes of Grasses (ISFEG 2015) and 1st International Symposium on Plant Microbiomes (ISPM).

[70] Vikuk V., Young C.A., Lee S.T., Nagabhyru P., Krischke M., Mueller M.J., Krauss J. (2019). Infection Rates and Alkaloid Patterns of Different Grass Species with Systemic *Epichloë* Endophytes. Appl. Environ. Microbiol..

[71] Bacon C.W., White J.F. (1994). Stains media and procedures for analyzing endophytes. Biotechnology of Endophytic Fungi of Grasses.

[72] White J.F., Morgan-Jones G., Morrow A.C. (1993). Taxonomy, Life Cycle, Reproduction and Detection of Acremonium Endophytes. Agric. Ecosyst. Environ..

[73] Simpson W.R., Faville M.J., Moraga R.A., Williams W.M., Mcmanus M.T., Johnson R.D. (2014). Epichloë Fungal Endophytes and the Formation of Synthetic Symbioses in Hordeeae (=Triticeae) Grasses. J. Syst. Evol..

[74] Hillis S.R.J. (2019). Transmission and Survival of Perennial Ryegrass Endophyte during Field Based Seed Production. Master’s Thesis.

[75] Groppe K., Boller T. (1997). PCR Assay Based on a Microsatellite-Containing Locus for Detection and Quantification of Epichloë Endophytes in Grass Tissue. Appl. Environ. Microbiol..

[76] Young C.A., Charlton N.D., Takach J.E., Swoboda G.A., Trammell M.A., Huhman D.V., Hopkins A.A. (2014). Characterization of Epichloë Coenophiala within the US: Are All Tall Fescue Endophytes Created Equal?. Front. Chem..

[77] Budowle B. (2014). Molecular Genetic Investigative Leads to Differentiate Monozygotic Twins. Investig. Genet..

[78] Hovermale J.T., Craig A.M. (2001). Correlation of Ergovaline and Lolitrem B Levels in Endophyte-Infected Perennial Ryegrass (*Lolium Perenne*). J. Vet. Diagn. Invest..

[79] Baldauf M.W., Mace W.J., Richmond D.S. (2011). Endophyte-Mediated Resistance to Black Cutworm as a Function of Plant Cultivar and Endophyte Strain in Tall Fescue. Environ. Entomol..

[80] Spiering M.J., Davies E., Tapper B.A., Schmid J., Lane G.A. (2002). Simplified Extraction of Ergovaline and Peramine for Analysis of Tissue Distribution in Endophyte-Infected Grass Tillers. J. Agric. Food Chem..

[81] Vassiliadis S., Elkins A.C., Reddy P., Guthridge K.M., Spangenberg G.C., Rochfort S.J. (2019). A Simple LC–MS Method for the Quantitation of Alkaloids in Endophyte-Infected Perennial Ryegrass. Toxins.

[82] Finch S.C., Prinsep M.R., Popay A.J., Wilkins A.L., Webb N.G., Bhattarai S., Jensen J.G., Hawkes A.D., Babu J.V., Tapper B.A. (2020). Identification and Structure Elucidation of Epoxyjanthitrems from Lolium Perenne Infected with the Endophytic Fungus Epichloë Festucae Var. Lolii and Determination of the Tremorgenic and Anti-Insect Activity of Epoxyjanthitrem I. Toxins.

[83] Soto-Barajas M.C., Zabalgogeazcoa I., González-Martin I., Vázquez-de-Aldana B.R. (2017). Qualitative and Quantitative Analysis of Endophyte Alkaloids in Perennial Ryegrass Using Near-Infrared Spectroscopy: NIRS to Detect *Epichloë* Alkaloids in Ryegrass. J. Sci. Food Agric..

[84] Logan C., Edwards G., Kerr G., Williams S. (2015). Ryegrass Staggers and Liveweight Gain of Ewe Lambs and Hoggets Grazing Four Combinations of Perennial Ryegrass and Strains of Endophyte. Proc. N. Z. Soc. Anim. Prod..

[85] Dimenna M.E., Mortimer P.H., Prestidge R.A., Hawkes A.D., Sprosen J.M. (1992). Lolitrem B Concentrations, Counts of *Acremonium Lolii* Hyphae, and the Incidence of Ryegrass Staggers in Lambs on Plots of *A. Lolii* -Infected Perennial Ryegrass. N. Z. J. Agric. Res..

[86] Rowan D.D., Garrick C.M.L., Bacon C.W., White J.F. (2018). Utilization of endophyte-infected perennial ryegrass for increased insect resistance. Biotechnology of Endophytic Fungi of Grasses.

[87] Chao A. (1981). On Estimating the Probability of Discovering a New Species. Ann. Stat..

[88] Kaur J., Ekanayake P.N., Tian P., Jong E.V.Z.D., Dobrowolski M.P., Rochfort S.J., Mann R.C., Smith K.F., Forster J.W., Guthridge K.M. (2015). Discovery and Characterisation of Novel Asexual Epichloë Endophytes from Perennial Ryegrass (Lolium Perenne L.). Crop Pasture Sci..

[89] Kliman R., Sheehy B., Schulyz J. (2008). Genetic Drift and Effective Population Size | Learn Science at Scitable. Nat. Educ..

[90] Eady C.C. (2018). Methods of Producing Cultivar Swards with Tailored Properties. U.S. Patent.

[91] Harvey I.C., Fletcher L.R., Emms L.M. (1982). Effects of Several Fungicides on the Lolium Endophyte in Ryegrass Plants, Seeds, and in Culture. N. Z. J. Agric. Res..

[92] Rolston M.P., Archie W.J., Simpson W.R. (2002). Tolerance of AR1 *Neotyphodium* Endophyte to Fungicides Used in Perennial Ryegrass Seed Production. N. Z. Plant Prot..

[93] Stewart A.V. (1986). Effect on the *Lolium* Endophyte of Nitrogen Applied to Perennial Ryegrass Seed Crops. N. Z. J. Exp. Agric..

[94] Rolston M.P., Hare M.D., Moore K.K., Christensen M.J. (1986). Viability of *Lolium* Endophyte Fungus in Seed Stored at Different Moisture Contents and Temperatures. N. Z. J. Exp. Agric..

[95] Hill N.S., Roach P.K. (2009). Endophyte Survival during Seed Storage: Endophyte-Host Interactions and Heritability. Crop Sci..

[96] Welty R.E. (1987). Influence of Moisture Content, Temperature, and Length of Storage on Seed Germination and Survival of Endophytic Fungi in Seeds of Tall Fescue and Perennial Ryegrass. Phytopathology.

[97] Hume D.E., Card S.D., Rolston M.P. Effects of Storage Conditions on Endophyte and Seed Viability in Pasture Grasses. Proceedings of the 22nd International Grasslands Congress.

[98] Gundel P.E., Garibaldi L.A., Martínez-Ghersa M.A., Ghersa C.M. (2011). Neotyphodium Endophyte Transmission to Lolium Multiflorum Seeds Depends on the Host Plant Fitness. Environ. Exp. Bot..

[99] Rolston M.P., Agee C. Delivery of quality seed to specification—The USA and NZ novel endophyte experience. Proceedings of the 6th International Symposium on Fungal Endophytes of Grasses.

[100] Arojju S.K., Cao M., Trolove M., Barrett B.A., Inch C., Eady C., Stewart A., Faville M.J. (2020). Multi-Trait Genomic Prediction Improves Predictive Ability for Dry Matter Yield and Water-Soluble Carbohydrates in Perennial Ryegrass. Front. Plant Sci..

[101] Do Canto J., Studer B., Frei U., Lübberstedt T. (2020). Pattern of Inheritance of a Self-fertility Gene in an Autotetraploid Perennial Ryegrass ( *Lolium Perenne* ) Population. Plant Breed..

[102] Zhang Y., Ran Y., Nagy I., Lenk I., Qiu J.-L., Asp T., Jensen C.S., Gao C. (2020). Targeted Mutagenesis in Ryegrass (Lolium Spp.) Using the CRISPR/Cas9 System. Plant Biotechnol. J..

[103] Christensen M.J., Bennett R.J., Ansari H.A., Koga H., Johnson R.D., Bryan G.T., Simpson W.R., Koolaard J.P., Nickless E.M., Voisey C.R. (2008). Epichloë Endophytes Grow by Intercalary Hyphal Extension in Elongating Grass Leaves. Fungal Genet. Biol..

[104] Spangenberg G.C., Guthridge K.M., Forster J., Sawbridge T.I., Ludlow E., Kaur J., Rochfort S.J., Rabinovich M., Ekanayake P.N. (2011). Endophytes and Related Methods. U.S. Patent.

[105] Australian Government (2019). Fungal Endophyte (Epichloe Festucae Var. Lolii). Aust. Gov. Plant Var. J..

[106] Muller H.J. (1964). The Relation of Recombination to Mutational Advance. Mutat. Res. Mol. Mech. Mutagen..

[107] Hettiarachchige I.K., Elkins A.C., Reddy P., Mann R.C., Guthridge K.M., Sawbridge T.I., Forster J.W., Spangenberg G.C. (2019). Genetic Modification of Asexual Epichloë Endophytes with the PerA Gene for Peramine Biosynthesis. Mol. Genet. Genom..

[108] Panaccione D.G., Johnson R.D., Wang J., Young C.A., Damrongkool P., Scott B., Schardl C.L. (2001). Elimination of Ergovaline from a Grass-Neotyphodium Endophyte Symbiosis by Genetic Modification of the Endophyte. Proc. Natl. Acad. Sci USA.

[109] Rahnama M., Forester N., Ariyawansa K.G.S.U., Voisey C.R., Johnson L.J., Johnson R.D., Fleetwood D.J. (2017). Efficient Targeted Mutagenesis in Epichloë Festucae Using a Split Marker System. J. Microbiol. Methods.

[110] Wang R. Abstract: CRISPR-Cas9 Knockout of the Epichloë Festucae Antifungal Protein Gene (ASA, CSSA and SSSA International Annual Meetings). Proceedings of the Managing Global Resources for a Secure Future.

[111] Schipper L.A., Mudge P.L., Kirschbaum M.U.F., Hedley C.B., Golubiewski N.E., Smaill S.J., Kelliher F.M. (2017). A Review of Soil Carbon Change in New Zealand’s Grazed Grasslands. N. Z. J. Agric. Res..

[112] Eady C., Courtney C. A pasture seed production perspective. New Zealand Institute of Agricultural and Horticultural Science AgScience 2021 57 18–19 Regenerative Agriculture Issue. https://indd.adobe.com/view/693a575a-5482-4df0-bc4d-f986d3bce648.

[113] Krauss J., Vikuk V., Young C.A., Krischke M., Mueller M.J., Baerenfaller K. (2020). Epichloë Endophyte Infection Rates and Alkaloid Content in Commercially Available Grass Seed Mixtures in Europe. Microorganisms.

[114] Reading K. Grass Named after Deputy PM Barnaby Joyce Recalled from Seed Market. https://www.abc.net.au/news/rural/2016-10-13/deputy-pm-barnaby-joyce-grass-recalled-toxic-fungus/7929322.

